# Intrafamilial variability of phenotype in *CACNA2D4-*associated retinal dysfunction: more or less

**DOI:** 10.1007/s10633-025-10047-w

**Published:** 2025-08-29

**Authors:** Vasily Smirnov, Claire-Marie Dhaenens, Vincent Canel, Sabine Defoort-Dhellemmes

**Affiliations:** 1 CHU Lille, Service d’Exploration de la Vision et de Neuro-Ophtalmologie, Hôpital Salengro, 59037 Lille, France; 2 U1172-LilNCog-Lille Neuroscience & Cognition, University Lille, Inserm, CHU Lille, 59045 Lille, France

**Keywords:** Stationary retinal dysfunction, CACNA2D4, Full field ERG, Intrafamilial variability

## Abstract

**Introduction:**

Retinal dysfunction associated with *CACNA2D4* gene defects is a rare disorder of photoreceptor to bipolar cell signaling. We report two affected siblings presenting a surprising disparity of retinal involvement.

**Materials and methods:**

Patients underwent complete ocular examination, multimodal fundus imaging, and full-field electroretinography (ffERG). Genetic testing was performed by a targeted Next Generation Sequencing panel.

**Results:**

Two siblings presented a reduced visual acuity and light sensitivity. ffERG was specific for *CACNA2D4*-related retinal dysfunction, but amplitudes of responses were different in the two patients. Additionally, an x-wave to a dim red flash was well preserved, and there was a reduced b/a ratio to a high intensity (30cd/m2) dark-adapted stimulus. Both patients were homozygous for the variant c.2406C > A, p.(Tyr802*) in *CACNA2D4.* During 17 years of follow-up, vision remained stable in patient 1, with no evidence of retinal degeneration.

**Conclusion:**

Intrafamily clinical and electrophysiological expression of *CACNA2D4-*associated retinal dysfunction can be variable.

**Supplementary Information:**

The online version contains supplementary material available at 10.1007/s10633-025-10047-w.

## Introduction

Biallelic gene defects in *CACNA2D4* (OMIM #608171) have been associated with autosomal recessive cone dystrophy (RCD4, OMIM #610478)[1], nonprogressive retinal dysfunction[2], and more recently with retinitis pigmentosa[3] and cone-rod dystrophy [4]. However, reports of these diseases are limited.

*CACNA2D4* encodes the α2δ4 subunit of photoreceptor voltage-gated L-type calcium channels (VGCC). VGCC is a multimeric protein localized in the presynaptic membrane of the photoreceptor-bipolar cell synapse. It triggers a calcium influx, contributing to a glutamate release and subsequent signal transmission from photoreceptors to bipolar cells [5, 6].

Here we describe two siblings harboring a homozygous *CANA2D4* gene defect, both presenting a nonprogressive retinal disorder. Despite this shared genetic variant, there is a surprising disparity in their clinical presentations and electroretinographic findings.

## Methods

A male patient and, more recently, his sister were examined. Clinical data, including best-corrected visual acuities (BCVA) measured with ETDRS chart at 4 m distance, orthoptic examination, slit lamp and dilated fundus examinations, were collected. Spectral-domain optical coherence tomography (SD-OCT) of the macula was performed with the Spectralis OCT (Heidelberg Engineering, Inc., Heidelberg, Germany). Fundus autofluorescence imaging (SWAF and NIRAF) was done with the Heidelberg Retinal Tomograph (Heidelberg Engineering, Inc., Heidelberg, Germany). Color fundus imaging was performed with a Zeiss Clarus 500 (Carl Zeiss Meditec AG, Jena, Germany).

Full-field electroretinogram (ffERG) was recorded using a MonColor® unit (Metrovision, Perenchies, France). The recording electrode was a Dencott® corneal contact lens placed after local anesthetic application (0.4% oxybuprocaine) and corneal protection by a 0.5% methylcellulose solution. The adhesive skin reference electrode was placed on the ipsilateral temple, and the ground electrode was positioned on the forehead. The pupils were dilated with 1% tropicamide and 10% phenylephrine. Standard stimulations (DA 0.01, 3, and 10, LA 3 and LA 30 Hz flicker) followed ISCEV recommendations [7]. Supplementary dark-adapted single-flash responses were also recorded with a 30 cd/m2 white stimulus. ON–OFF ERG was obtained with broadband white stimulus (70 cd s/m2) of 100 ms duration on a white background (30 cd/m2)[8]. A dim red flash dark-adapted ERG was obtained with a 0.02 cd s/m2 red stimulus of 640 nm [9].

Genetic assessment was performed using a large NGS panel including 230 inherited retinal dystrophy genes, including *CACNA2D4* [10]. Capture oligonucleotide probes were designed using the Haloplex target enrichment System (Agilent Technologies Inc., Santa Clara, CA, USA). DNA libraries were sequenced on a NovaSeq sequencer (Illumina Inc., San Diego, CA, USA). The data analysis was conducted using an in-house developed pipeline compiling the data obtained from Seqnext (JSI Medical System, Ettenheim, Germany) and GATK software. Variants in *CACNA2D4* were confirmed by bidirectional Sanger sequencing of the exon 25 and exon–intron boundaries (NM_172364.5). The primer sequence is available on demand. Patient 2 underwent supplementary testing on ocular malformation NGS panel [11], including in particular *SLC38A8* and *PAX6*.

## Results

A male patient of 13 y.o. (patient 1) was initially referred for a reduced visual acuity despite no apparent eye disease. He complained of light adaptation issues: transitioning from dark/dim environments to bright light, he experienced a temporary loss of vision, describing himself as “apparently blind” for several seconds. Using his smartphone upon awakening, the text appeared blurred and distorted. He did not report any night blindness complaints. His past medical history was otherwise unremarkable.

**Fig. 1:**
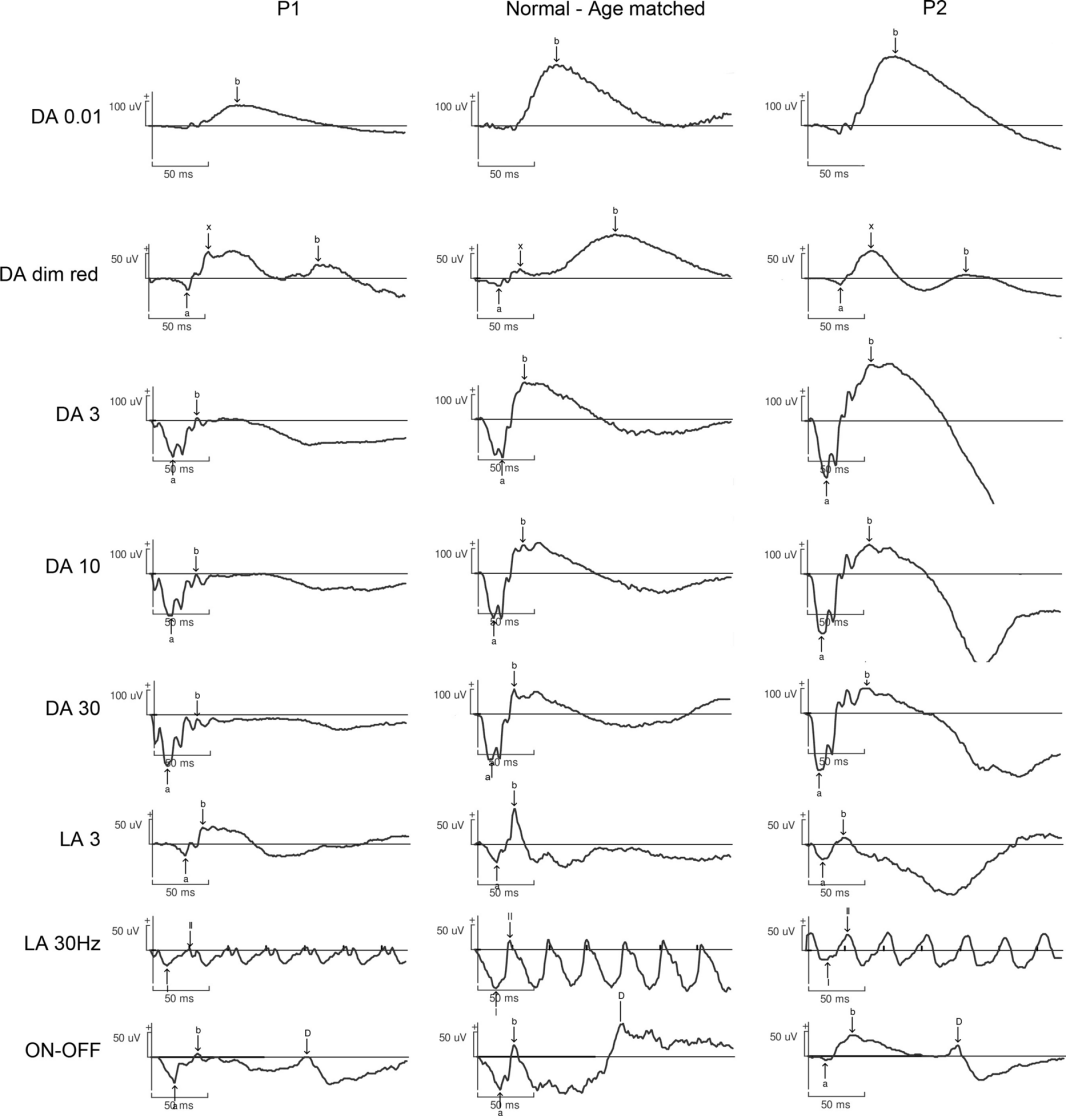
Full field ERG. *P1—patient 1.* Reduced amplitude of dark-adapted responses (DA 0.01, DA 3, DA 10). Well-preserved x-wave to DA dim red stimulus, reduced b/a ratio in DA 30. Reduced amplitude of light-adapted responses, multiphasic b-wave with flattened descending limb to LA 3*. P2—patient 2.* Supranormal amplitudes of dark-adapted responses. Multiphasic b-wave. Reduced light-adapted responses. Note the difference in the amplitude of responses in P1 and P2

**Fig. 2:**
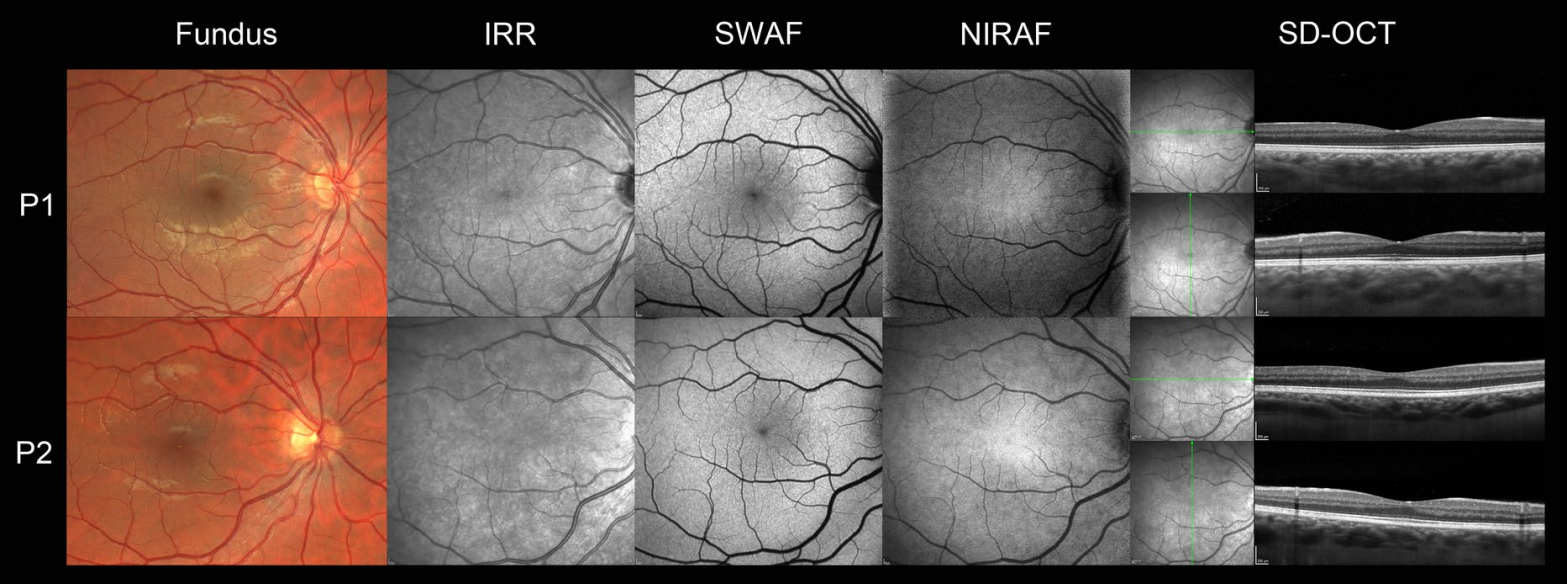
Multimodal retinal imaging. *Top row, patient 1.* Normal appearance of fundus, IRR, SWAF, NIRAF, and SD-OCT. *Bottom row, patient 2*. Grade 1 foveal hypoplasia. No degenerative retinal changes were found in both patients

BCVA was 20/40 in each eye with + 3.50 (− 0.75)160° RE and + 3.0 LE cycloplegic spectacle correction. There was no strabismus nor nystagmus. Slit lamp examination of anterior segment and fundus were unremarkable. Ishihara plates, Farnsworth, and Lanthony 15 Hue ranking tests were all normal. Static and kinetic visual fields were normal. Under dark-adapted conditions, ffERG (Fig. 1, P1) discovered a reduced b-wave to DA 0.01, reduced a and b-waves to DA 3 and DA 10 stimuli. Curiously, dark-adapted dim red-flash ERG depicted a well-preserved x-wave and a severely reduced b-wave. There was an exaggerated visibility of OPs on the ascending limb of the b-wave and a supplementary reduction of the b/a ratio with stimulus intensity increment (DA 30). Light-adapted responses were more severely reduced for both a and b-waves. The aspect of LA 3 b-wave was also peculiar, multiphasic. It was flattened and delayed for the descending limb, giving the overall aspect of a mountain-shaped b-wave. LA 30 Hz flicker was of pseudo-cycloid form with an abrupt descending limb of each phase. ON–OFF ERG responses were reduced in amplitude, but all the components were present. Multimodal retinal imaging, including color fundus photo, IRR, SWAF, NIRAF, and HD-OCT, was normal (Fig. 2, top row).

Patient 1 was re-assessed at ages 16, 18, and finally at 30 y.o. There was no change in his visual symptoms. BCVA was 20/32 with + 1.50 spectacle correction OU at last examination. Visual fields, color vision tests, ffERG, and multimodal imaging depicted no changes from the first assessment (Supplementary Data).

His elder sister asked to assess her vision because of symptoms similar to those of her brother. She (patient 2 of 35 y.o.) reported the same issues of light adaptation and mild light sensitivity from her teen years. BCVA was 20/25 in each eye and 20/20 both eyes with + 2.0 spectacle correction. She was orthophoric with no nystagmus and a normal stereopsis. Visual fields and color vision tests (including 100-Hue Farnsworth-Munsell test) were normal. Slit lamp and fundus examination were unremarkable. As for the ffERG (Fig. 1, P2), the amplitudes of dark-adapted responses were supranormal with no b/a ratio reduction at higher intensity stimuli. Light-adapted responses were reduced in amplitude and depicted the same morphological abnormalities as in patient 1. On multimodal retinal imaging, there was no evidence of retinal degeneration, but there was a grade I foveal hypoplasia (Fig. 2, bottom row).

Both patients were siblings of a French caucasian family. Parents denied consanguinity. NGS sequencing, realized 17 years after the initial clinical assessment in patient 1, revealed a homozygous variant in *CACNA2D4*: c.2406C > A, p.(Tyr802*), which was already reported[1]; no structural variants were detected. This variant was classified as pathogenic (Ia: PVS1, PS3, PM2, PP1-M) according to ACMG criteria [12]. All rare variants found by NGS and not associated with disease are listed in Supplementary Data, Table 1. Afterwards, Sanger sequencing of *CACNA2D4* exon 25 was performed for patient 2 and revealed the same homozygous variant. Due to associated foveal hypoplasia, patient 2 was tested for *SLC38A8* and *PAX6* gene defects: none were detected.

## Discussion

We described 2 siblings in a French nonconsanguineous family with a stationary retinal dysfunction linked to *CACNA2D4* gene defect. Patient 1 was more severely affected: he had lower BCVA compared to his sister (Patient 2) and thus had been diagnosed earlier. Light sensitivity and prolonged light adaptation pointed to a predominant photopic dysfunction.

Multimodal imaging did not reveal any retinal degeneration in both patients. No degenerative changes appeared on retinal imaging during the 17-year follow-up in patient 1 (Fig. 2 and Supplementary). These facts pinpoint a nonprogressive character of the disease, as previously suggested [2]. Interestingly, patient 2 presented a low-degree foveal hypoplasia, but she was free of nystagmus and had full stereopsis. No pathogenic variants have been identified in major genes associated with isolated foveal hypoplasia (*PAX6* and *SLC38A8*).

The diagnosis was suspected given peculiar ERG abnormalities, predominant under light-adapted conditions. As previously reported, dark-adapted responses were reduced in amplitude but more preserved than light-adapted responses. LA 3 ERG depicted a distinctive multiphasic b-wave. Once again, all ERG responses were more altered in patient 1 compared to patient 2 (Fig. 1). Such intrafamilial variability has never been reported in two siblings harboring the same *CACNA2D4* variant [1]. Phenotypic variability is a major concern for rare disorders, as it may lead to delayed diagnosis [13]. Both environmental and genetic modifiers have been reported to influence this variability. In dominant disorders, cis- and trans-acting genetic modifiers can either enhance or suppress the expression of the pathogenic allele [14]. In contrast, the mechanisms underlying recessive disorders differ, and genetic modifiers may involve genes encoding protein partners within the same functional pathway [15]—in this case, the retinal signaling pathway. Various approaches are recommended for studying genetic interactions [13]; however, this aspect is beyond the scope of the current work.

Additionally, there was an electronegative response configuration to DA 30 in patient 1, not observed in patient 2(Fig. 1). Noteworthy, the first patients with *CACNA2D4*-associated retinal disease were retrieved from a group with an electronegative ERG and initial diagnosis of night blindness [1]. In a spontaneously occurring murine model, the ERG was electronegative as well [16]. Dim red flash dark-adapted ERG in both siblings showed a well-preserved and even supranormal x-wave, pointing to a better preservation of dark-adapted cone function (Fig. 1, DA dim red). We hypothesized that the electronegative DA 30 configuration may be linked to a photopic hill phenomenon, specific to cone-driven responses under dark-adapted conditions [17]. Also, there were no ON–OFF ERG alterations (Fig. 1, ON–OFF) specific for Schubert-Bornschein complete and incomplete forms of congenital stationary night blindness [18]. Thus, we considered the ERG of patient 1 as pseudo-electronegative. BaAbad et al. suggested that expression of *CACNA2D4* is not essential for normal rod function [2].

Two reports suggested that *CACNA2D4* biallelic missense variants could be associated with retinitis pigmentosa. Patient harboring c.955G > A p.(Asp319Asn) and c.1822A > C p.(Ile608Leu) complained of increased need for light, had a nystagmus and an oculomotor apraxia [3], which were not the features of our patients. Another patient harboring the homozygous c.2120G > A, p.(Arg707His) was reported with cone-rod dystrophy [4]. The variant identified in our patients, c.2406 C > A p.(Tyr802*)*,* has already been reported in two patients with similar ERG findings [1]. Homozygous c.1882C > T p.(Arg628*) and c.1719 + 2904_2551-27827del35862insTG p.(?) variants were also associated with non-progressive retinal dysfunction phenotype [2]. These three latter are loss-of-function variants predicted to be responsible for non-functional protein or absence of protein synthesis.

*CACNA2D4* encodes the α2δ4 accessory subunit of VGCC, which modulates the signaling by increasing channel presence on the cell membrane and also by influencing its activation/inactivation kinetics [19]. In *Cacna2d4* knock-out (KO) mouse model, patch-clamp recordings on photoreceptors revealed a substantial reduction in Ca^2+^ influx. There was also a disorganization (immature size) and number reduction of rod ribbons. Cone synaptic terminals were less affected morphologically, but there was a reduction of cone VGCC function [20]. Interestingly, patch-clamp recordings on bipolar cells (BCs) showed a reconfiguration of retinal circuitry with rod contribution to cone-BCs response and abolishment of normal rod-to-rod-BCs signaling under dark-adapted conditions [20]. This fact may explain a supranormal x-wave to DA dim red stimuli in our patients. However, mouse phenotype does not recapitulate the human one and is closer to congenital stationary night blindness [16, 20, 21]. By contrast, a cone-dominated zebrafish KO model mimicked the mildly impaired cone function of patients and the slowly or nondegenerative nature of the defect [22].

In conclusion, we reported two siblings with a non-progressive retinal dysfunction associated with *CACNA2D4* biallelic nonsense variant. The clinical and electrophysiological expression of the disease varied in this sibship.

## Supplementary Information

Below is the link to the electronic supplementary material.

Supplementary file1 (DOCX 536 KB)
