# Supplementary material for: Intrafamilial variability of phenotype in CACNA2D4-associated retinal dysfunction: more or less
Source: Doc Ophthalmol. 2025 Aug 29;151(3):271–7. doi: 10.1007/s10633-025-10047-w (PMC12568804; doi:10.1007/s10633-025-10047-w)
Supplement: Supplementary file 1 — Supplementary file1 (DOCX 536 KB) [file 10633_2025_10047_MOESM1_ESM.docx]

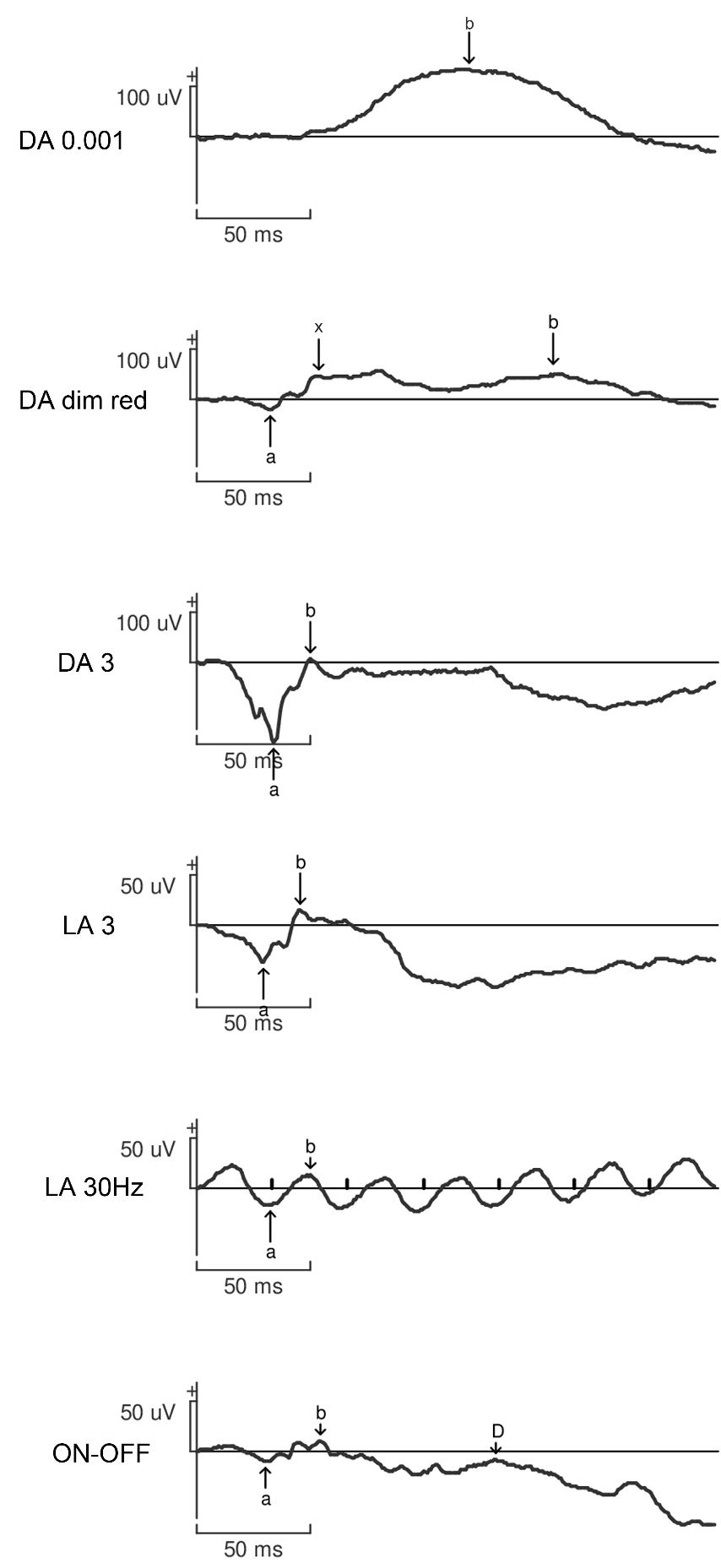


**Supplementary Figure 1.** ffERG ot the patient 1 at 13 y.o. Same ffERG appearance as at 30 y.o. (compare with Fig.1 in the main text)


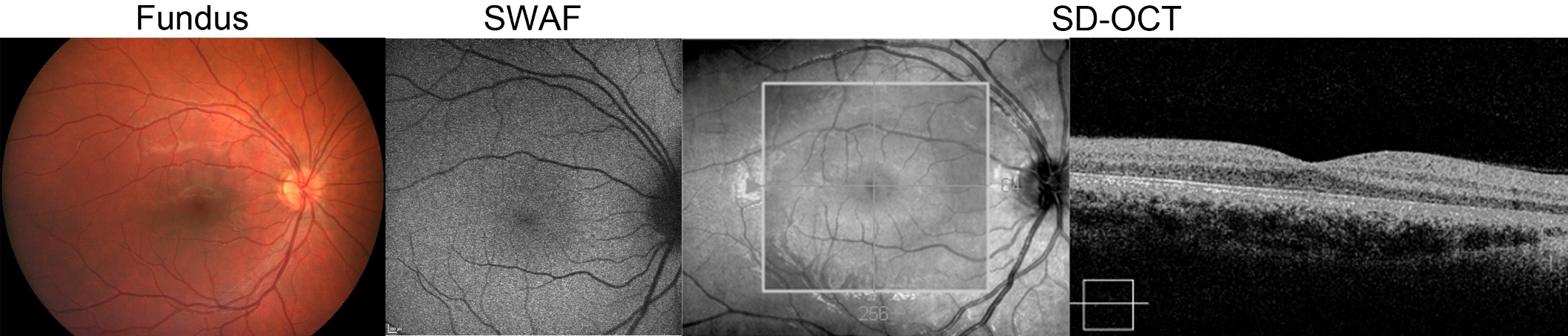


**Supplementary Figure 2.** Multimodal retinal imaging of the patient 1 at 13 y.o. No degenerative retinal changes. Same appearance as at 30 y.o. (compare with Fig.2 in the main text).

**Suppelmentary Table 1**. Rare variants not associated with disease found in Patient 1.

| **Chr** | **Position** | **Gene** | **Transcript** | **Nucleotide change** | **Amino acid change** | **Consequence** | **ACMG**  **classification** |
| --- | --- | --- | --- | --- | --- | --- | --- |
| 16 | 77325391 | *ADAMTS18* | NM_199355 | c.3190-16C>G |  | intron_variant | Class 1 |
| 6 | 135716008 | *AHI1* | NM_017651 | c.3015A>G | p.Ser1005= | synonymous_variant | Class 1 |
| 16 | 56535386 | *BBS2* | NM_031885 | c.1104C>T | p.Asn368= | synonymous_variant | Class 2 |
| X | 49082499 | *CACNA1F* | NM_005183 | c.1556G>A | p.Arg519Gln | missense_variant | Class 1 |
| X | 49071522 | *CACNA1F* | NM_005183 | c.3630+24C>T |  | intron_variant | Class 1 |
| **12** | **1953632** | ***CACNA2D4*** | **NM_172364** | **c.2406C>A** | **p.Tyr802Ter** | **stop_gained** | **Class 5** |
| 11 | 117263809 | *CEP164* | NM_014956 | c.2583G>A | p.Val861= | synonymous_variant | Class 2 |
| 11 | 117267807 | *CEP164* | NM_014956 | c.3279C>T | p.Ser1093= | splice_region_variant&  synonymous_variant | Class 2 |
| 20 | 34057799 | *CEP250* | NM_007186 | c.936A>G | p.Thr312= | synonymous_variant | Class 1 |
| 16 | 3582896 | *CLUAP1* | NM_015041 | c.1092+55A>T |  | intron_variant | Class 1 |
| 21 | 46925127 | *COL18A1* | NM_130445 | c.2948G>A | p.Arg983His | missense_variant | Class 1 |
| 16 | 72143406 | *DHX38* | NM_014003 | c.3474G>C | p.Arg1158= | synonymous_variant | Class 1 |
| 6 | 66063346 | *EYS* | NM_001142800 | c.1459+5C>T |  | splice_region_variant&  intron_variant | Class 2 |
| 1 | 186089112 | *HMCN1* | NM_031935 | c.12095-27dup |  | intron_variant | Class 2 |
| 1 | 185931632 | *HMCN1* | NM_031935 | c.1829-18G>A |  | intron_variant | Class 1 |
| 16 | 1569871 | *IFT140* | NM_014714 | c.4040+11G>A |  | intron_variant | Class 1 |
| 6 | 76715263 | *IMPG1* | NM_001563 | c.888-12T>G |  | intron_variant | Class 1 |
| 9 | 139327581 | *INPP5E* | NM_019892 | c.1159+26C>T |  | intron_variant | Class 1 |
| 9 | 139327577 | *INPP5E* | NM_019892 | c.1159+30T>A |  | intron_variant | Class 1 |
| 18 | 7000030 | *LAMA1* | NM_005559 | c.4383-34T>A |  | intron_variant | Class 1 |
| 4 | 128861133 | *MFSD8* | NM_152778 | c.573A>G | p.Thr191= | synonymous_variant | Class 1 |
| 5 | 149242788 | *PDE6A* | NM_000440 | c.2400C>T | p.Asp800= | synonymous_variant | Class 1 |
| 5 | 149247637 | *PDE6A* | NM_000440 | c.2199+21A>C |  | intron_variant | Class 1 |
| 4 | 651291 | *PDE6B* | NM_000283 | c.1401+8G>A |  | splice_region_variant&  intron_variant | Class 2 |
| 4 | 651287 | *PDE6B* | NM_000283 | c.1401+4C>T |  | splice_region_variant&  intron_variant | Class 1 |
| 19 | 54627281 | *PRPF31* | NM_015629 | c.681G>A | p.Thr227= | synonymous_variant | Class 1 |
| 9 | 116053115 | *PRPF4* | NM_004697 | c.1256+28G>A |  | intron_variant | Class 1 |
| 17 | 63221214 | *RGS9* | NM_003835 | c.1502G>A | p.Arg501His | missense_variant | Class 1 |
| 2 | 234224738 | *SAG* | NM_000541 | c.93G>A | p.Gly31= | synonymous_variant | Class 1 |
| 1 | 156146546 | *SEMA4A* | NM_022367 | c.2044C>T | p.Pro682Ser | missense_variant | Class 1 |
| 1 | 156131109 | *SEMA4A* | NM_022367 | c.811-28C>G |  | intron_variant | Class 2 |
| 15 | 31342586 | *TRPM1* | NM_002420 | c.1371+26G>A |  | intron_variant | Class 1 |
| 16 | 49856589 | *ZNF423* | NM_015069 | c.8A>C | p.Lys3Thr | missense_variant | Class 2 |
| 16 | 49672834 | *ZNF423* | NM_015069 | c.278-49C>T |  | intron_variant | Class 1 |
| 2 | 27601843 | *ZNF513* | NM_144631 | c.290C>T | p.Ala97Val | missense_variant | Class 1 |
|  |  |  |  |  |  |  |  |
| *Variants were considered as rare if frequency in the patients cohort analyzed with this NGS pipeline is <1%* | | | | | | |  |
